# Study on Occupational Allergy Risks (SOLAR II) in Germany: Design and methods

**DOI:** 10.1186/1471-2458-11-298

**Published:** 2011-05-11

**Authors:** Sabine Heinrich, Astrid Peters, Jessica Kellberger, Diana Ellenberg, Jon Genuneit, Dennis Nowak, Christian Vogelberg, Erika von Mutius, Gudrun Weinmayr, Katja Radon

**Affiliations:** 1 Unit for Occupational and Environmental Epidemiology & Net Teaching, Institute and Outpatient Clinic for Occupational, Social and Environmental Medicine, University Hospital of Munich (LMU), Munich, Germany; 2 Paediatric Department, University Hospital of Dresden, Dresden, Germany; 3 Institute of Epidemiology, University of Ulm, Ulm, Germany; 4 Dr. v. Haunersches Kinderspital, University Hospital of Munich (LMU), Munich, Germany

## Abstract

**Background:**

SOLAR II is the 2^nd ^follow-up of a population-based cohort study that follows the participants of ISAAC Phase Two recruited in Munich and Dresden in 1995/6. A first follow-up study was conducted 2002 and 2003 (SOLAR I). The aims of SOLAR II were to investigate the course of atopic diseases over puberty taking environmental and occupational risk factors into account. This paper describes the methods of the 2^nd ^follow-up carried out from 2007 to 2009 and the challenges we faced while studying a population-based cohort of young adults.

**Methods:**

Wherever possible, the same questionnaire instruments were used throughout the studies. They included questions on respiratory and allergic diseases, domestic and occupational exposure and work related stress. Furthermore, clinical examinations including skin prick tests, spirometry and bronchial challenge with methacholine, exhaled nitric oxide (FeNO) and blood samples were employed at baseline and 2^nd ^follow-up. As information from three studies was available, multiple imputation could be used to handle missing data.

**Results:**

Of the 3053 SOLAR I study participants who had agreed to be contacted again, about 50% had moved in the meantime and had to be traced using phone directories and the German population registries. Overall, 2904 of these participants could be contacted on average five years after the first follow-up. From this group, 2051 subjects (71%) completed the questionnaire they received via mail. Of these, 57% participated at least in some parts of the clinical examinations. Challenges faced included the high mobility of this age group. Time constraints and limited interest in the study were substantial. Analysing the results, selection bias had to be considered as questionnaire responders (54%) and those participating in the clinical part of the study (63%) were more likely to have a high parental level of education compared to non-participants (42%). Similarly, a higher prevalence of parental atopy (e.g. allergic rhinitis) at baseline was found for participants in the questionnaire part (22%) and those participating in the clinical part of the study (27%) compared to non-participants (11%).

**Conclusions:**

In conclusion, a 12-year follow-up from childhood to adulthood is feasible resulting in a response of 32% of the baseline population. However, our experience shows that researchers need to allocate more time to the field work when studying young adults compared to other populations.

## Background

Asthma and allergies are multi-factorial diseases with genetic and environmental risk factors. Birth cohort studies have given important insights into risk factors in early life contributing to these diseases during childhood [1-5], but only few of them so far followed participants until adulthood [4-7], and none of them took occupational risk factors into account. This might change as current birth cohorts, recently summarized by Keil and colleagues, reach adult age [6,7]. However, many of these cohorts are high-risk cohorts and it is thus not easy to extrapolate their results to the general population [6,7].

Cohort studies starting in adulthood have shown that occupational exposure is associated with new onset and aggravation of respiratory and atopic diseases [8-10]. Of these, only the European Community Respiratory Health Survey (ECRHS) was done at the general population level with sufficient case numbers to identify occupational risk factors prospectively [8,11]. Based on this multicentre study, the population-attributable risk of occupational exposure for adult asthma is estimated to be 10 to 25% [12]. However, information on childhood disease, childhood exposure and the course of disease over puberty is limited, because this study started at adult age. Therefore, it is difficult to distinguish new onset and recurrence of disease in this study.

In 2002 and 2003, we therefore conducted a first follow-up of the then 16 to 18-year-old participants of the International Study of Asthma and Allergies in Childhood (ISAAC) Phase Two in Munich and Dresden [13,14].

The ISAAC Phase Two study described the regional variation in the prevalence of atopic diseases in children using standardized approaches that were implemented accurately. In the first follow-up called "Study on Occupational Allergy Risks (SOLAR) I", 3785 participants of ISAAC Phase Two completed a standardized questionnaire that included data on respiratory symptoms and environmental exposure [15-17]. The results of this study already indicated at this point that the occupational contribution to the new onset and persistence of symptoms during the first months of employment was considerable [15,16,18] and that atopic teenagers did not take their diseases into account when selecting a job [19].

At the time of the first follow-up many of the participants still attended secondary school and occupational exposure was mainly confined to holiday jobs. Thus, a second follow-up was planned for 2007 to 2009. This second follow-up also included clinical examinations in order to get in-depth information about the respiratory health and allergic sensitization of the participants.

The aims of SOLAR II were:

• to describe the course of respiratory diseases and atopy from childhood over puberty to young adulthood in a relatively large population-based sample;

• to identify environmental risk factors and their importance for the course of respiratory and atopic diseases;

• to assess the association between stress and the course of the diseases under study;

• to identify occupational risk factors for the course of these diseases.

Using the results of this study we would like to improve career counselling in adolescents with respiratory or atopic diseases. Furthermore, we are interested in identifying markers of respiratory and atopic diseases with an onset at an early stage during vocational training, that is soon after the beginning of occupational exposure.

This paper presents the methods and data concerning study participation as well as non-responder analyses of SOLAR II. The paper will help to facilitate planning and performance of future studies investigating the natural course of diseases in adolescent cohorts.

## Methods

### Study design

**Figure 1:**
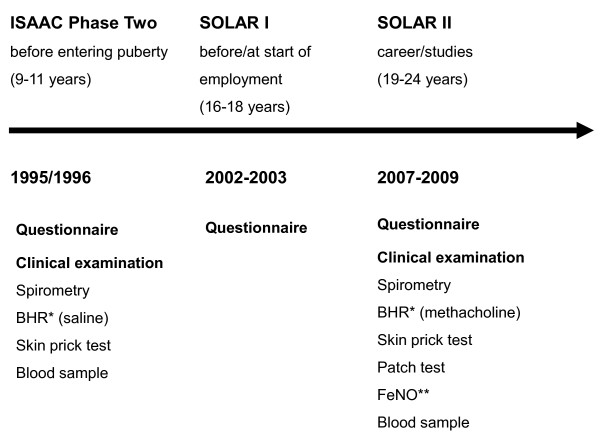
**Flow-chart of the study**. * BHR = examination of bronchial hyperresponsiveness. ** FeNO = measurement of exhaled nitric oxide.

An overview of the study design is given in Figure 1.

#### Baseline study: ISAAC Phase Two

The German part of the multicentre International Study of Asthma and Allergies in Childhood (ISAAC) Phase Two investigated the prevalence of allergies and asthma in grade 4 pupils (age 9-11 years) in Munich and Dresden in 1995-1996. Details of this study have been described elsewhere [13,20]. Parents of 7498 children were asked to complete a questionnaire on demographic, health and environmental factors. 85% of the parents contacted filled in the questionnaire (table 1).

**Table 1:** Participation and reasons for non-participation in ISAAC Phase Two, SOLAR I and SOLAR II

	Total	Munich	Dresden
	n (%)	n (%)	n (%)

**ISAAC Phase Two** Questionnaire study	6399 (85.3)^1^	3354 (87.6)	3045 (83.0)

**SOLAR I**	3785 (77.4)^2^	2043 (81.5)	1742 (73.0)
of these:			
Agreed to be re-contacted	3053 (80.7)	1534 (75.1)	1519 (87.2)

**SOLAR II**			
**Drop-outs in total**	**149 (4.9)**	**85 (5.5)**	**64 (4.2)**
			
Address could not be found via population registries	143 (4.7)	82 (5.3)	61 (4.0)
Deceased	6 (0.2)	3 (0.2)	3 (0.2)
**Eligible study population**	**2904 (100.0)**	**1449 (100.0)**	**1455 (100.0)**
**Refusals in total**	**853 (29.4)**	**441 (30.4)**	**412 (28.3)**
of these			
*Could not be reached by phone/no phone number available*	454 (53.3)	184 (41.7)	270 (65.5)
*Lack of interest/time*	262 (30.7)	146 (33.0)	116 (28.2)
*Absence from study location during the entire field phase*	59 (6.9)	41 (9.3)	18 (4.4)
*Other reasons*	78 (9.1)	70 (15.8)	8 (1.9)
**Response: questionnaire part**	**2051 (70.6)**	**1008 (69.6)**	**1043 (71.1)**
**Response: clinical study**	**1167 (40.2)**	**568 (39.2)**	**599 (41.2)**

^1 ^6399 of 7498 invited children

^2 ^3785 of 4893 invited adolescents who could be re-contacted

In addition, a random sample of these children was selected and tested for asthma symptoms using spirometry and for bronchial hyperreactivity using nebulised hypertonic saline. Allergic sensitization was tested by skin prick tests and specific IgE in blood serum. Atopic dermatitis was assessed by standardized skin examinations.

#### First follow-up: SOLAR I

SOLAR I (Study on Occupational Allergy Risks I, 2002-2003) was the first follow-up investigating ISAAC Phase Two participants whose parents had agreed to be contacted again later. At the time of the survey participants were 16 to 18 years old. SOLAR I mainly focused on the question whether there was an association between starting the working life and the course of atopic and respiratory diseases [12,21].

Overall, 4893 respondents (77%) could be re-contacted. Of these, 3785 adolescents (77%) took part and gave informed consent to combine the baseline and follow-up data (table 1). The questionnaire included 118 validated items on respiratory health, allergies, preferred job choices, smoking, environmental tobacco smoke exposure, jobs including holiday jobs and vocational training, environmental risk factors and chronic stress. Most items were taken from the European Community Respiratory Health Survey (ECRHS) and the International Study of Asthma and Allergies in Childhood (ISAAC) [12,21]. Wherever possible the same items as in ISAAC Phase Two were employed. Each of the preferred job choices and of the jobs carried out so far was coded independently by two trained coders according to the International Standard Classification of Occupations-88 code (ISCO-88 system) (International Labour Office 1999). Potential occupational exposure was assigned using an asthma-specific job-exposure matrix (JEM) [22].

#### Second follow-up: SOLAR II

All SOLAR I participants who agreed to be re-contacted and whose address could be traced were invited to take part in a questionnaire study and clinical examinations from 2007 to 2009. At that time participants were 19 to 24 years old.

All phases of the study were approved by the Ethical Committees of the Medical Faculty of the University of Dresden, the Bavarian Chamber of Physicians and the University of Ulm.

### Recruitment methods

All address data of participants who agreed to be re-contacted (n = 3053) were checked first by electronic phone directory and - if no address data could be found (about 50%) - by the local population registries. In Germany, all citizens are required by law to register their address with the local registration office. If somebody moves, e.g. from one town to another, the population registry of the previous place of living receives information about where the citizen has moved. Furthermore, children's current home addresses are always registered with their parents' addresses. This way, 95% of respondents could be retraced. The remaining participants of SOLAR I had either moved abroad or had not registered with the local registration office.

We sent an invitation consisting of an information letter, an informed consent form, the SOLAR II questionnaire and an invitation to take part in the clinical examination to the participants' current addresses. Additionally, a ball pen with the SOLAR logo was added to increase participation [23]. In order to ensure that the clinical examination could be done within eight weeks after the questionnaire response was received, 100-150 questionnaires per study centre were sent out every month. This way, we wanted to achieve consistency between questionnaire response and clinical examination results. In addition, participation in the clinical part was assumed to be higher.

In order to maximize response, non-responders received up to two postal reminders and up to five reminder phone calls. Those who verbally refused to participate were asked to answer an 11-item short-questionnaire to assess selection bias and to obtain information about their reasons for refusal.

Those who agreed to participate in the clinical examination were contacted by phone within seven days after receiving the questionnaire and were offered an appointment at the local study centre. If a phone number was available, those who answered the questionnaire but did not indicate participation in the clinical examination were contacted by the local study physician and asked for their reasons for non-participation. This way, many people who initially did not want to participate changed their mind after the doctor explained the procedures.

A large proportion of them initially refused, because they lived far away from the local study centre (e.g., because they attended university in a different town). They were given an appointment at the local study centre for their next visit to their home town. Others were afraid of certain examinations (e.g. bronchial provocation) and did not know that it was possible to participate only in certain parts of the examinations.

### Questionnaire instruments

The 133-item questionnaire focused on

• socio-demographic data (3 items)

• respiratory symptoms and diseases (35 items)

• hand eczema and atopic dermatitis (13 items)

• domestic exposure (17 items)

• smoking, environmental tobacco smoke (7 items)

• occupation (27 items):

◦ level of education

◦ job choice and use of occupational counselling

◦ job history for all jobs held for at least one month and for at least eight hours a week

◦ occupational diseases

◦ occupational risk factors

• physical activity, body mass index (4 items)

• body height and weight, oral contraceptives, number of pregnancies (5 items)

• work related stress (TICS, Trier Inventory of the Assessment of Chronic Stress) (22 items) [24].

Wherever possible, items identical to those employed in SOLAR I and ISAAC Phase Two were used. In comparison to SOLAR I, questions concerning family status, number of children, hand eczema and occupational exposure to gas, fumes, cleaning agents etc. were added. Additional items were taken from the ECRHS, the GA^2^LEN survey [25] and the TICS [24].

During the design of the SOLAR II questionnaire, the third version of TICS already existed. Unfortunately, in comparison to the first TICS version, which had been used in SOLAR I, the wording of the items and the subscales had changed to some extent. To guarantee comparability of the data with the questionnaire data of SOLAR I, the scales "work overload" and "dissatisfaction" of the first version were used again and the new scale "pressure to succeed" of the third version was added.

Participants' job histories since SOLAR I were coded independently by two trained coders according to the ISCO-88 system (International Labour Office 1999). Afterwards an expert re-evaluation step was carried out. Exposure to agents with potential asthma risk was assessed by means of an asthma-specific job-exposure matrix (JEM) [22].

### Clinical examinations

**Figure 2:**
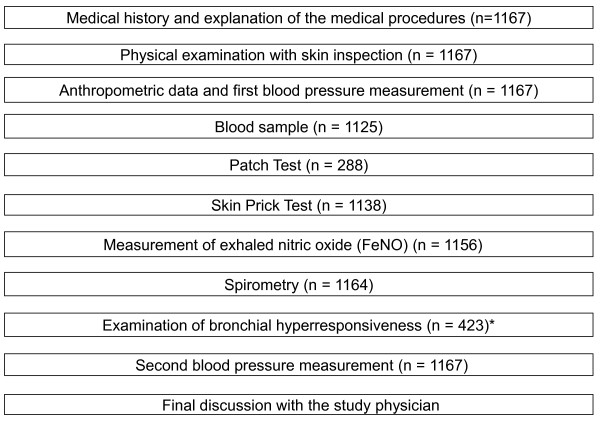
**Steps of a regular medical examination in SOLAR II**. * Examination of bronchial hyperresponsiveness was only offered to 576 participants who also underwent spirometry in ISAAC Phase Two, 423 of these took part.

Clinical examinations as described in Figure 2 were offered to all participants. In each study centre, a team of one study physician and one study nurse carried out all parts of the clinical examinations. These were scheduled as requested by the participants. Therefore, they were offered at various times of the day. Weekend appointments were also offered but were not well accepted by the participants; very few of the scheduled participants actually showed up to their weekend appointments. One or two days before the medical examination the participants were contacted by phone or per text message to remind them of their appointment.

After the medical procedures were explained by the study physician, the participant and the study physician signed the informed consent form. Although the physicians encouraged the participants to take part in all parts of the examinations (Figure 2), the participants could refuse parts of the examinations e.g. due to health reasons such as pregnancy. Information on pregnancy and birth was collected using the pregnancy certificate of the participants' mothers.

Including bronchial provocation, the clinical examinations took approximately 1.5 hours per participant. At the end of the examinations a report with all individual results was printed and explained to the participant by the study physician. If these results indicated pathological findings the participants were encouraged to consult their general practitioner. In addition, participants received a shopping voucher as reimbursement.

At baseline (ISAAC Phase Two) and 2^nd ^follow-up (SOLAR II) the clinical examinations included skin prick tests, spirometry, bronchial challenge and blood samples. Spirometry and bronchial hyperresponsiveness were performed according to the ECRHS II protocol. According to the recommendations for children bronchial hyperresponsiveness was performed using hypertonic saline (0,9%) in ISAAC Phase Two. In SOLAR II, for the now adult participants, we converted to methacholine according to official recommendations. Skin prick tests were performed according to the ISAAC Phase Two protocol. In ISAAC Phase Two, total serum IgE levels were measured using the Imulite System (DPC Biermann, Bad Nauheim, Germany). In SOLAR II, total serum IgE levels were measured with Fluoroenzymimmunoassay using UniCAP 100 (Phadia).

#### Anthropometric data

Body weight and height as well as hip and waist size were measured in all participants with calibrated instruments. Additionally, blood pressure was measured with identical automatic devices (OMRON M5-1, OMRON HEALTHCARE Europe B.V., Hoofddorp, the Netherlands) in Dresden and Munich to avoid device-specific discrepancies. Blood pressure was assessed at least once after a defined resting period to avoid artificial hypertension. If possible, blood pressure was measured again at the end of the examination.

#### Blood samples

Blood samples were collected to measure total immunoglobulin E (IgE) and α_1_-antitrypsin and to perform a multi-allergen test for occupational allergens (professional allergen mix (PAX 5) consisting of TDI, MDI and HDI isocyanates and phthalic anhydride). Serum samples were stored at -20°C and then transferred to the laboratory of the University Hospital of Munich (LMU) where the analyses were performed. Additionally, according to standardized procedures, blood samples with anticoagulant EDTA for genetic analyses regarding asthma-related genes were taken from those participants who gave additional informed consent to this procedure.

#### Skin prick test

The skin prick tests (SPT) were conducted following the ISAAC Phase Two protocol [20]. Exclusion criteria were pregnancy and breast-feeding. Participants were asked not to use any medication that could possibly affect the result of the SPT such as antihistamines. If participants used such medication, this was noted in the medical database with the time of the last intake.

In ISAAC Phase Two, control solutions (histamine and physiological saline solution^1^) and extracts of common aeroallergenes (Dermatophagoides pteronyssinus^1^, D. farinae^1^, cat^1^, Alternaria tenius^1^, mixed grass pollen^1 ^and mixed tree pollen) were used. In SOLAR II, occupational allergens (rye flour^1^, rat^1^, mouse^3^, *Lepidoglyphus destructor*-Lep D 1^2^, tyrophagus^2^, aspergillus^1^, Alpha-amylase^4 ^and latex^4^) as well as rye pollen^1 ^and common ragweed^1 ^were added. As mixed tree pollen was out of production, the study group decided to investigate the three types of tree pollen (birch^1^, hazel^1^, alder^1^) separately. The solutions were produced by ^1^ALK Scherax (Wedel, Germany), ^2^Allergopharma (Reinbek, Germany), ^3^Bencard Allergie (Munich, Germany) and ^4^Alyostal Pricktest of Stallergenes GmbH (Kamp-Lintfort, Germany).

The test evaluation was performed using the positive control as reference parameter in order to avoid a possible inter- and intra-rater bias. A reaction was defined as positive if its wheal size (average of the longest diameter and the midway perpendicular diameter) was at least half the size of the positive control [26]. SPT results were entered into the medical database. Multiple training sessions were performed for the clinical staff to ensure the validity and reliability as suggested by the ISAAC Phase Two protocol [20]. This was done in order to guarantee that the same method was applied in both study centres.

#### Exhaled nitric oxide (FeNO)

The fraction of exhaled nitric oxide (FeNO) was analysed in both centres using the analyser CLD 88 sp (ECO MEDICS AG, Duernten, Switzerland) with the DENOX 88 device and the SPIROWARE software. The analyser was calibrated at the beginning of each study day. For each participant at least three FeNO levels were recorded.

#### Spirometry

For the lung function measurements, participants were asked to avoid smoking for one hour prior to the examination, refrain from ß2-agonists or anticholinergic inhalers for four hours before the examination and not to use oral medications (ß2-agonists, theophylline, antimuscarinics) at least eight hours before the test. Participants reporting an infection of the respiratory system within three weeks prior to the clinical examination were re-scheduled. If this was not possible, the medication or infection was documented in the clinical database.

Lung function was measured using the MasterScope^® ^spirometer (Viasys Health Care GmbH, formerly JAEGER, Würzburg-Höchstadt, Version 4.53). The measurements were performed and evaluated according to American Thoracic Society criteria [27].

In short, measurements were performed in sitting position with the subject wearing a nose clip. For each participant at least three technically satisfactory manoeuvres should be recorded. If it was not possible to obtain at least three technically satisfactory manoeuvres after nine attempts, lung function testing was stopped. Reference values of Quanjer et al. were used to evaluate the results [28]. Obstruction was defined as a FEV_1 _value <80% predicted.

#### Bronchial hyperresponsiveness to methacholine

Bronchial hyperresponsiveness (BHR) was tested using APS nebulizers (Viasys Health Care) according to the adapted ECRHS protocol for a stepwise methacholine challenge [29,30]. The ISAAC Phase Two protocol was not suitable for the SOLAR II age group. As mentioned earlier, only those participants who performed a spirometry in ISAAC Phase Two were asked to take part in BHR testing in SOLAR II in order to assess the long-term course. Doubled or quadrupled doses of methacholine were used until a drop in FEV_1 _of 20% occurred. However, in contrast to the ECRHS protocol, the maximum cumulative dose used was limited to 0.6 mg; we have shown earlier that higher doses might lead to an overestimation of bronchial hyperresponsiveness [31].

#### Skin examination and patch test

The skin of all participants was examined for atopic dermatitis and hand eczema. Therefore, the predilection areas in the face and the flexural folds on arms and hands were examined in detail. If the participants showed skin symptoms, digital photography was used for documentation and the affected skin areas were registered in the medical database.

Starting from November 2007, additional patch tests were applied. The tests were conducted with the commercial TRUE Test™ (Mekos Laboratories ApS, Denmark) panels I and II consisting of 24 contact allergens (table 2). Additionally, ten primary occupational contact allergens (BRIAL, Greven, Germany) were tested in all participants (table 2). Participants were asked to remove the test layers after 48 hours and to renew the markers that surrounded the patches in order to facilitate test interpretation. Exclusion criteria were pregnancy, no testable area of skin in the area of the upper back (e.g. due to eczema or acne), a previous patch test or a known contact allergy to one of the used allergens.

**Table 2:** Allergen panels of the patch test

Panel I^1^	Panel II^2^	Panel III^3^
Nickel sulphate	Butylphenol formaldehyde resin	Ammonium persulfate

Wool alcohols	Paraben mix	P-Toluenediamine

Neomycin sulphate	Carba mix	Monoethanolamine

Potassium dichromate	Black rubber mix	Diethanolamine

Caine mix	Cl+Me-Isothiazolinone	P-Aminoazobenzene

Fragrance mix	Quaternium-15	N, 'N-Methylene-bis-5-methyl-oxazolidine

Colophony	Mercaptobenzothiazole	Zinc-dibutyldithiocarbamate

Epoxy resin	P-Phenylenediamine	Glyoxal trimer (dihydrate)

Quinoline mix	Formaldehyde	Glutardialdehyde

Balsam of Peru	Mercapto mix	Bioban CS-1135

Ethylenediamine dihydrochloride	Thiomersal	

Cobalt chloride	Thiuram mix	

^1 2 ^TRUE Test™ (Mekos Laboratories ApS, Denmark)

³ BRIAL, Greven, Germany

Skin reactions were checked only once after approx. 72 hours for feasibility reasons, as participants were not willing to visit the study centre three times. This is common practice in many epidemiological studies. Even with this current approach, participants often refused to take part in the patch test due to time constraints and the strict requirements for a successful testing (no water on the testing area, no sports, etc. for 72 hours). The skin reactions were labelled according to the recommendations of the International Contact Dermatitis Research Group [32]. In short, questionable and irritant reactions were counted as negative reactions.

### Quality control procedures

A pilot study was conducted in both centres with 35 young adults to evaluate the comprehensibility of the questions and the time required to complete the questionnaire. Questions of the ISAAC Phase Two and the SOLAR I survey were not changed to guarantee the comparability between studies. Other questions were adapted if the participants had difficulties understanding their meaning.

Adherence to the study protocol was ensured through training workshops with the field staff of the two study centres at the beginning of and on regular basis throughout the field phase. All technical and clinical equipment was calibrated regularly.

### Data management

Questionnaire data were entered at the data centre at Ulm University by an automatic data capture system (Teleform Desktop V9.1 and Teleform Workgroup V10.1, Autonomy Cardiff, Vista, CA, USA) into an MS ACCESS database. Plain text entries were entered manually. In addition to automatic alert, specific items were re-evaluated by two trained data entry clerks. Information on the recruitment of the participants and response as well as data from the clinical examinations were entered into MS ACCESS databases. The resulting data-sets were merged to the previous studies (ISAAC Phase Two and SOLAR I) using a unique identifier. Data quality was checked routinely and included global checks on participant mismatches from various data sources and plausibility checks.

### Imputation of missing data

Missing data were assumed to be missing at random (MAR), thus allowing for application of multiple imputation [33]. Two different methods were used for the imputation of five respective data sets:

a) using the AMELIA II package in R

b) drawing from the empirical distribution of the variables of interest.

In both cases, imputation was restricted to missing exposure and confounding questionnaire data. 98% of the variables imputed had a low missingness of less than 14% (range 0.1%-21.9%) of the data.

### Statistical analyses

Initially, data were analysed descriptively. Bivariate associations between potential confounders and outcomes were assessed as well as possible bivariate associations between exposure and outcomes. Furthermore, longitudinal statistical models concerning different aspects of occupational exposure and diseases were fitted. We analysed whether pre-existing symptoms had influenced the job choice of the participants. Moreover, individual risk factors and probabilities for the incidence of occupational asthma and allergies in certain job groups were estimated. The latency period between start of (occupational) exposure and the first symptoms was taken into account when reconsidering the best time for a follow-up.

### Non-responder analyses

To evaluate potential selection bias, ISAAC Phase Two results were compared for three groups of participants:

• Group 1: people who participated only in the ISAAC Phase Two questionnaire survey

• Group 2: people who participated in the ISAAC Phase Two and SOLAR I, but not in SOLAR II

• Group 3: people who participated in the ISAAC Phase Two, SOLAR I and SOLAR II questionnaire survey

## Results

### Participation

Overall, 3053 young adults were contacted and asked to participate in SOLAR II. For 143 people no recent home address could be obtained from the population registries, six people had died. Therefore, 2904 people were eligible for the study. 2051 people (71% of the eligible sample) answered the questionnaire (table 1).

853 of the remaining participants of SOLAR I (29% of the eligible sample) refused to take part in SOLAR II. The most frequent reason for non-participation was that the subjects did not answer the invitation letter, that no telephone number was available or they could not be reached by phone (455 people; 53%) (table 1). Only 30 of the non-responders were willing to fill in the short non-responder questionnaire.

In Munich, 56% and in Dresden 57% of those who completed the SOLAR II questionnaire took part in the clinical examinations (40% of the eligible sample) (table 1). Of the 884 participants (30% of the eligible sample) who filled in the questionnaire but refused to take part in the clinical examinations, 26% did not want to participate due to lack of interest and 19% due to time constraints. A further 12% no longer lived close to the study centres and thus felt unable to participate.

### Non-responder analyses

ISAAC Phase Two results were compared for three groups of participants. Children of parents with higher socio-economic status were more likely to participate in the follow-up studies. In addition, respiratory symptoms or disease in ISAAC Phase Two and parental atopy as well as the lack of passive smoke exposure during childhood and no smoking of the mother during pregnancy were predictors for participation in SOLAR I and II (table 3).

**Table 3:** Non-responder analysis: Descriptive results and prevalence of atopic diseases, data taken from ISAAC Phase Two.

% 95% CI^1^	Group 1^2^ (n = 2581)	Group 2^3^ (n = 1767)	Group 3^4^ (n = 2051)
Female	55.0 53.1; 56.9	53.8 51.5; 56.1	58.1 56.0; 60.3

High parental level of education^5^	**41.5** **39.5; 43.5**	**47.1** **44.7; 49.4**	**58.9** **56.7; 61.0**

Smoking during pregnancy	**13.5** **11.9; 15.1**	**9.8** **8.3; 11.4**	**5.9** **4.8; 7.0**

Passive smoke exposure	50.3 48.3; 52.4	**47.3** **44.9; 49.7**	**36.4** **34.3; 38.5**

Current wheeze^6^	**6.9** **5.9; 7.9**	**9.5** **8.1; 10.8**	8.2 7.0; 9.4

Doctor diagnosed asthma	3.3 2.6; 4.0	4.5 3.5; 5.5	3.4 2.6; 4.2

Current allergic rhinitis^7^	**11.1** **9.8; 12.3**	**14.1** **12.4; 15.7**	14.4 12.8; 15.9

Current atopic dermatitis^8^	**11.6** **10.4; 12.9**	13.2 11.6; 14.8	**15.2** **13.6; 16.7**

Specific IgE > 0.7 kU/L	**28.7** **26.0; 31.3**	**34.8** **32.1; 37.4**	33.4 31.0; 35.7

Positive skin prick test	23.2 21.0; 25.5	24.5 22.3; 26.7	23.6 21.7; 25.6

Bronchial hyperreactivity	14.7 11.9; 17.4	19.5 16.5; 22.4	17.3 14.8; 19.8

Parental allergic disease^9^	**36.2** **34.3; 38.1**	**40.9** **38.6; 43.3**	**46.2** **44.0; 48.3**

^1 ^95% CI: 95% Confidence Interval

^2 ^group 1: people who participated only in ISAAC Phase Two

^3 ^group 2: people who participated in ISAAC Phase Two and SOLAR I

^4 ^group 3: people who participated in SOLAR II (without the people of group 1 and 2)

^5 ^at least one parent with a minimum of 12 years of education

^6 ^wheezing in the last 12 months

^7 ^problems with sneezing or a runny or blocked nose (without having a cold) accompanied by itchy-

watery eyes in the last 12 months

^8 ^itching eczema in the last 12 months persisting at least 6 months

^9 ^asthma, allergic rhinitis or atopic dermatitis

**bold**: statistically significant differences between groups

Participants with a higher level of education, students, people with a higher parental educational level and participants in occupations with high-risk exposure of asthma were more likely to participate in the clinical examinations. Smokers and people with regularly passive smoke exposure were less motivated. However, participation in the clinical examination was independent of parental atopy, respiratory symptoms of the young adults or a current occupation in the health care sector (table 4).

**Table 4:** Non-responder analysis: SOLAR II questionnaire data compared for participants of the questionnaire part and the clinical part of the study

% 95% CI^1^	Questionnaire part only (n = 884)	Questionnaire and clinical part (n = 1167)
Female	56.3 53.1; 59.6	59.5 56.6; 62.3

High level of education^2^	**52.5** **49.1; 55.8**	**70.0** **67.4; 72.7**

High parental level of education^3^	**54.2** **50.8; 57.5**	**62.4** **59.6; 65.2**

Student	**36.3** **33.1; 39.4**	**50.5** **47.6;53.3**

Current occupation: health care sector	13.1 10.9; 15.4	14.7 12.7; 16.8

Smoker	**44.0** **40.7; 47.3**	**32.0** **29.3; 34.7**

Passive smoke exposure (regularly)	**63.3** **60.1; 66.5**	**51.5** **48.6;54.3**

Job with high-risk exposure^4^	**25.8** **22.5;29.2**	**32.3** **29.2; 35.3**

Job with low-risk exposure^4^	23.0 19.8; 26.2	19.9 17.3; 22.5

Job without risk exposure^4^	36.3 32.6; 39.9	39.7 36.5; 42.8

Current wheeze^5^	16.3 13.9; 18.8	17.2 15.1; 19.4

Doctor diagnosed asthma	8.3 6.4; 10.1	9.3 7.6; 10.9

Current allergic rhinitis^6^	22.1 19.3; 24.9	27.0 24.4; 29.5

Current atopic dermatitis^7^	11.3 9.2; 13.4	13.1 11.1; 15.0

Parental allergic disease^8^	42.9 39.7; 46.2	48.6 45.7; 51.5

^1 ^95% CI: 95% Confidence Interval

^2 ^at least 12 years of education

^3 ^at least one parent with a minimum of 12 years of education

^4 ^exposure to agents with potential asthma risk was assessed by means of an asthma-specific job-

exposure matrix (JEM)

^5 ^wheezing in the last 12 months

^6 ^problems with sneezing or a runny or blocked nose (without having a cold) accompanied by itchy-

watery eyes in the last 12 months

^7 ^itching eczema in the last 12 months persisting at least 6 months

^8 ^asthma, allergic rhinitis or atopic dermatitis

**bold**: statistically significant differences between groups

## Discussion

We are presenting the study design and the first results of a prospective population-based cohort study investigating atopic and respiratory diseases among young adults in Germany aged 19 to 24 years today. The main focus of SOLAR II is on the association between occupational exposure and the course of atopic and respiratory diseases.

One of the strengths of this study is its adequate sample size with follow-up data for over 2000 young adults and the follow-up period of more than 12 years. Therefore, and thanks to the prospective study design, detailed information about job history, occupational exposure and persistence, recurrence or new-onset of atopic diseases could be assessed.

Furthermore, multiple imputation could be used to handle missing data due to the availability of information from three studies. In addition, data from ISAAC Phase Two and SOLAR I make it possible to estimate selection bias in the analyses.

Another advantage of SOLAR II is the detailed assessment of a number of exposure and diseases using standardized and validated protocols for the questionnaire and the clinical examination [20] as well implementing a quality control. Additionally, several arrangements were made to increase participation as far as possible and to decrease selection bias. These arrangements included postal reminders, up to five telephone calls, text messages as reminders of the clinical examinations, flexible examination hours and shopping vouchers for participation in the clinical examination.

Investigating the age group of young people and young adults, it's important also to use the "new" communication technologies which are very popular among these age groups. One or two days before the medical examination the participants were contacted by phone or per text message as reminder of their fixed date. We also collected the email addresses of the participants to send them a route description (if they took part in the medical examination) and a message concerning the main results of the study. Therefore, if a third follow-up study would take place, it would be possible to use an online questionnaire.

Selection bias had to be considered in the result analyses, as females and children of parents with higher socio-economic status were more likely to participate in the follow-up studies. This issue had already arisen during SOLAR I. In addition, parental atopy and respiratory symptoms or disease in ISAAC Phase Two were predictors of participation in SOLAR I and II. People without passive smoke exposure during childhood and participants whose mothers didn't smoke during pregnancy were also more likely to participate in the follow-up studies. Probably, children of health conscious parents are more motivated to participate.

Therefore, a selection towards higher educated participants with higher likelihood of atopic diseases can be assumed. The higher level of education might lead to lower risk of occupational exposure. This may result in a selection bias if participation was differential with respect to exposure and disease. I.e., if participants with higher exposure were more likely to be symptomatic while those with lower exposure were less likely to be symptomatic, an overestimation of the true risk may result. This needs to be taken into consideration when interpreting the results.

Concerning the interpretation of the results of the clinical examination of SOLAR II, it should be considered that participants with a higher level of education, students, people with a high parental education level and people in occupations with high-risk exposure of asthma were more likely to participate. Presumably, students had less time problems due to their flexible time-table and long semester breaks as well as a higher interest to take part in a scientific study. The number of smokers and people with regularly passive smoke exposure was higher among the participants who only filled in the questionnaire.

## Conclusions

In conclusion, a 12-year follow-up from childhood to adulthood is feasible resulting in a response of 32% of the baseline population. However, our experience shows that researchers need to allocate more time to the field work when studying young adults compared to other populations. For example, about 50% of the participants' addresses needed to be checked by the local population registries. Furthermore, postal reminders, reminder phone calls and in some cases second or even third appointments for the clinical examination were necessary to achieve adequate response. These results could be helpful for birth cohorts that now reach adulthood.

## Abbreviations

BHR: Bronchial hyperresponsiveness; ECRHS: European Community Respiratory Health Survey; FeNO: exhaled nitric oxide; GA^2^LEN: Global Allergy and Asthma European Network; IgE: total immunoglobulin E; ISAAC Phase Two: International Study of Asthma and Allergies in Childhood Phase Two; ISCO-88 system: International Standard Classification of Occupations-88 code; JEM: job-exposure matrix; SOLAR: Study on Occupational Allergy Risks; SPT: skin prick test; TICS: Trier Inventory of the Assessment of Chronic Stress;

## Competing interests

The authors declare that they have no competing interests.

## Authors' contributions

SH was one of the principle investigators responsible for design, coordination, acquisition of data and writing the manuscript. AP was also one of the principle investigators responsible for coordination and acquisition of data. JK was also one of the principle investigators and responsible for acquisition of data, data analysis and interpretation of the data. DE was responsible for acquisition of data. JG made contributions to conception and design and was responsible for data management. DN made contributions to conception and design and also to analyses and interpretation of the data. CV made contributions to conception and design and was also responsible for coordination and acquisition of data. EvM made contributions to conception, design and analyses. GW made contributions to conception and design and was also responsible for data management. KR made contributions to conception and design and also to analyses, interpretation of the data and drafting the manuscript. All authors read and approved the final manuscript.

## Pre-publication history

The pre-publication history for this paper can be accessed here:

http://www.biomedcentral.com/1471-2458/11/298/prepub
